# Repetition and Emotive Communication in Music Versus Speech

**DOI:** 10.3389/fpsyg.2013.00167

**Published:** 2013-04-04

**Authors:** Elizabeth Hellmuth Margulis

**Affiliations:** ^1^Music Cognition Lab, Department of Music, University of ArkansasFayetteville, AR, USA

**Keywords:** repetition, basal ganglia, sequencing, speech-to-song illusion, ritual

## Abstract

Music and speech are often placed alongside one another as comparative cases. Their relative overlaps and disassociations have been well explored (e.g., Patel, 2008). But one key attribute distinguishing these two domains has often been overlooked: the greater preponderance of repetition in music in comparison to speech. Recent fMRI studies have shown that familiarity – achieved through repetition – is a critical component of emotional engagement with music (Pereira et al., 2011). If repetition is fundamental to emotional responses to music, and repetition is a key distinguisher between the domains of music and speech, then close examination of the phenomenon of repetition might help clarify the ways that music elicits emotion differently than speech.

## Music’s Repetitiveness is Special

Ethnomusicologist Nettl (1983) identifies musical repetition as a rare cultural universal – a characteristic exhibited by the music of every known human culture. Although some traditions, for example certain strands of contemporary art music in the West, explicitly eschew repetition, they do so in conscious response to a tendency toward musical repetition that exists elsewhere in the culture. Evolutionary biologist Fitch (2006) goes so far as to call repetition a “design feature” of music, essentially constitutive of the communicative form. This repetition can happen within a piece, or across multiple hearings.

Speech, by contrast, features a much lower incidence of repetition, and although the specifics are challenging to quantify, aspects of this distinction are plainly evident. For example, music features a litany of symbols instructing the player to repeat, from repeat signs to da capo indications (Kivy, 1993), whereas written language possesses no such lexicon for repetition. In a plea to abolish the practice of “part-repetition,” a tradition in eighteenth century music whereby performers would repeat large sections of the piece during performance, Ferdinand Praeger appeals to the unpalatability such a practice would have in speech:

Would ever a poet think of repeating half of his poem; a dramatist a whole act; a novelist a whole chapter? Such a proposition would be at once rejected as childish. Why should it be otherwise with music? … Since any whole part-repetition in poetry would be rejected as childish, or as the emanation of a disordered brain, why should it be otherwise with music? (Praeger, 1882–1883).

Yet the fact remains not only that sections within musical pieces are often repeated, but also that entire pieces are listened and relistened to hundreds of times, often voluntarily and even enthusiastically.

Garcia (2005) explores the ways that repetition’s perceived affiliations with childishness, regression, and insanity (well exemplified by Praeger’s remarks) have prevented scholars from acknowledging, let alone investigating its function in music (with notable exceptions, such as Ockelford, 2005). They’ve preferred instead to emphasize music’s connections with language, long recognized as a legitimate domain of inquiry. But insight into the parallels between music and language has sometimes come at the cost of insight into music’s more unique qualities, like repetitiveness. So closely affiliated with music is this quality, in fact, that its use within speech can actually serve to engender a perceptual shift whereby an acoustic stimulus first perceived as speech comes to be perceived as music. This phenomenon, the speech-to-song illusion (Deutsch et al., 2011), documents the way that the temporally regular repetition of a particular clause can trigger a startling effect on replay of the entire utterance whereby the speaker, at the start of the clause in question, is heard to suddenly break into song. That the simple act of repetition can so dramatically musicalize speech illuminates its special role in delineating these two communicative domains.

## Are there Functional Commonalities Underlying Different Kinds of Repetition?

Johnstone’s (1994) two-volume edited collection explores a variety of special cases where language is used repetitively, asking fundamentally whether there are things “repetition always does” (p. 12). By way of an answer, Johnstone observes that:

The function of repetition in general is to point, to direct a hearer back to something and say, “Pay attention to his again. This is still salient; this still has potential meaning; let’s make use of it in some way.” This accounts, for example, for the cognitive utility of repetition to learners, getting the learner’s attention on a token of input for a second round in order to have something to work with. We can also call attention to the fact that we’re getting one’s attention, and we can take that one step further, when awareness of the ability to manipulate allows us to play with attention. Immediacy may be poetic. … Repetition is a mode of focusing attention. … Repetition focuses attention on the makeup of both the repeated discourse and the earlier discourse. Repetition puts the utterance in brackets making it impossible to treat the language as if it were transparent, by forcing hearers to focus on the language itself. In that sense repetition is metalinguistic (p. 13).

Repetition in speech, in other words, encourages a listener to orient differently to the repeated element, to shift attention down to its constituent sounds on the one hand, or up to its contextual role on the other. For example, if a mob boss in a gangster movie says “take care of it,” and is answered by a quizzical look, he might repeat “take care of it!” underscoring for his henchman the darker meaning of the instruction.

The speech-to-song illusion can be understood similarly as a shift to a different level of understanding, in this case, to the utterance’s lower-level prosodic aspects. Semantic satiation (Severance and Washburn, 1907), the well-known phenomenon whereby repeatedly speaking a word causes it to shed its semantic associations and devolve into nonsense, can also be understood as a result of an attentional shift down to the word’s lower-level phonemic content.

Recent work in music has suggested that in addition to engendering a downward shift, repetition can also trigger an attentional shift up, toward progressively higher levels of the musical structure (Margulis, 2012). When participants were reexposed to the same piece four times in a row and asked during each iteration to press a button each time they heard something repeat, having been previously informed that the repeating thing could range in scope from a two-note motive to a phrase or section, they generally identified repetitions of smaller-scale elements (such as motives) on the first hearing, and then progressively larger-scale elements (such as phrases) across additional exposures. This change can be interpreted as evidence of a shift in orientation from lower-level aspects of the musical structure to higher-level aspects of the musical structure. Although repeated exposures seemed to engender an attentional shift upward for these pieces, I hypothesize that for repertoires with less rich hierarchic structuring repeated exposures might push attention down to attributes like microtiming and microdynamics.

In speech, then, repetition may be useful in specialized circumstances where a speaker wants a listener to attend to some different, non-obvious aspect of the utterance: its previously unseen relevance to some larger situational context, for example, or its prosodic or lexical content. But speech normally functions to relay a particular semantic meaning; once the message has been conveyed, the particular words used to convey it are no longer relevant. This condition has been explored within the context of the fuzzy trace theory (Reyna and Brainerd, 1995), which posits a distinction between gist and verbatim memory. Speech is normally associated with gist memory; if asked to recount a story, for example, people use different words to describe the events – they’ve invested in the *meaning* rather than in the particular words used to encode it. Music, by way of contrast, is associated with comparatively keener verbatim memory (Calvert, 1991, 2001). Recent work by Krumhansl (2010) shows that listeners can identify songs remarkably well from clips shorter than half a second, suggesting extremely acute verbatim encoding. Music doesn’t serve as a discardable vessel for conceptual meaning in the same way that ordinary uses of speech can; rather, its surface, verbatim content retains communicative significance across repetitions. Moving up and down within its structure across rehearings can yield satisfying varieties of engagement with a piece, revealing a stark contrast in the kind of thing sought after by a listener when hearing a piece of music versus an excerpt of speech.

## Repetition and Emotional Response

To gain a foothold in the relatively underexplored domain of the emotional impact of musical repetition, it’s helpful to examine a better-explored domain that features repetitive behavior, such as ritual. Like music, ritual features unusual degrees of voluntarily undertaken repetition, and also like music, ritual is capable of eliciting strong emotional response. Boyer and Liénard (2006) adopt a framework for event hierarchies from Zacks et al. (2001) to characterize the special behaviors associated with ritual. Within this framework, gestures (on the order of a few seconds) combine to form episodes (such as tying your shoes) which combine in turn to form scripts (such as getting ready for school or eating dinner at a restaurant). It’s typically most natural to recall events in terms of episodes, and excessive focus on the lower gestural level can indicate pathologies such as frontal lobe damage or schizophrenia (Janata and Grafton, 2003). But ritual expressly drives attention down to this level, inducing, Boyer and Liénard claim, a special mental state focusing on low-level properties of actions. Associated with the repeated gestures comes a general effect of goal demotion, where the uses the gestures are typically put to recede and the constituent movements themselves rise to prominence. The excessive repetition also serves as a powerful signal of intentionality, revealing both the internal commitment of the ritual’s participant and her ties to a social community that has defined these particular gestures as significant. Shifts in attention, then, of the sort chronicled by the studies reviewed in the first part of this article, might underlie the capacity for a special kind of emotional engagement.

Margulis (2013) arbitrarily inserted repetitions into excerpts of contemporary art music by renowned composers Elliott Carter and Luciano Berio, and everyday listeners without special training or experience with the genre rated the repetition-hacked examples as more likely to have been composed by a human artist and the original versions as more likely to have been randomly generated by a computer. Repetition in music, like repetition in ritual, then, can serve to signal intentionality, and this recognition of intentionality might facilitate the capacity to engage with sounds as emotionally communicative.

## Internal Imagery, External Sound, and Strong Experiences of Music

One consequence of the prevalence of musical repetition is the phenomenon of the earworm. Liikkanen (2008) reports that over 90% of people report experiencing earworms at least once a week, and more than 25% say they suffer them several times a day. Brown (2006), a neuroscientist at McMaster, has reported extensively on his own “perpetual music track:” tunes loop repeatedly in his mind on a near constant basis. Brown observes that the snippets usually last between 5 and 15 s, and repeat continuously – sometimes for hours at a time – before moving to a new segment.

The experience in each of these cases, the earworm and the perpetual music track, is very much one of being occupied by music, as if a passage had really taken some kind of involuntary hold on the mind, and very much one of relentless repetitiveness. The seat of such automatic routines is typically held to be the basal ganglia (Boecker et al., 1998; Nakahara et al., 2001; Lehéricy et al., 2005). Graybiel (2008) has identified episodes where neural activity within these structures becomes locked to the start and endpoints of well-learned action sequences, resulting in a chunked series that can unfurl automatically, leaving only the boundary markers subject to intervention and control. Vakil et al. (2000) showed that the basal ganglia underlie sequence learning even when the sequences lack a distinct motoric component. And, critically, Grahn and Brett (2007, 2009) used neuroimaging to demonstrate the role of the basal ganglia in listening to beat-based music; (Grahn and Rowe, 2012) shows that this role relates to the active online prediction of the beat, rather than the mere *post hoc* identification of temporal regularities.

The circuitry that underlies habit formation and the assimilation of sequence routines, then, also underlies the process of meter-based engagement with music. And it is repetition that defines these musical routines, fusing one note to the next increasingly tightly across multiple iterations. DeBellis (1995) offers this telling example of the tight sequential fusing effected by familiar music: ask yourself whether “oh” and “you” are sung on the same pitch in the opening to *The Star-Spangled Banner*. Most people cannot answer this question without starting at the beginning and either singing through or imagining singing through to the word “you.” We largely lack access to the individual pitches within the opening phrase – we cannot conjure up a good auditory image of the way “you” or “can” or “by” sounds in this song, but we can produce an excellent auditory image of the entire opening phrase, which includes these component pitches. The passage, then, is like an action sequence or a habit; we can duck in at the start and out at the end, but we have trouble entering or exiting the music midphrase. This condition contributes to the pervasiveness of earworms; once they’ve gripped your mind, they insist on playing through until a point of rest. The remainder of the passage is so tightly welded to its beginning that conscious will cannot intervene and apply the brakes; the music spills forward to a point of rest whether you want it to or not.

Reencountering a passage of music involves repeatedly traversing the same imagined path until the grooves through which it moves are deep, and carry the passage easily. It becomes an overlearned sequence, which we are capable of executing without conscious attention. Yet in the case of passive listening, this movement is entirely virtual; it’s evocative of the experience of being internally gripped by an earworm, and this parallel forms a tantalizing link between objective, external and subjective, internal experience. This sense of being moved, of being taken and carried along in the mode of a procedural enactment, when the knowledge was presented (by simply sounding) in a way that seemed to imply a more declarative mode can be exhilarating, immersive, and boundary-dissolving: all characteristics of strong experiences of music as chronicled by Gabrielsson and Lindström’s (2003) survey of almost 1000 listeners. Most relevant to the present account are findings that peak musical experiences tended to resist verbal description, to instigate an impulse to move, to elicit quasi-physical sensations such as being “filled” by the music, to alter sensations of space and time, including out-of-body experiences and percepts of dissolved boundaries, to bypass conscious control and speak straight to feelings, emotions, and senses, to effect an altered relationship between music and listeners, such that the listener feels penetrated by the music, or merged with it, or feels that he or she is being played by the music, to cause the listener to imagine him or herself as the performer or composer, or experience the music as executing his or her will, and to precipitate sensations of an existential or transcendent nature, described variously as heavenly, ecstatic or trance-like.

These sensations can be parsimoniously explained as consequences of a sense of virtual inhabitation of the music engendered by repeated musical passages that get procedurally encoded as chunked sequences, activating motor regions and getting experienced as lived/enacted phenomena, rather than heard/cognized ones. It is repetition, specifically, that engages and intensifies these processes, since it takes multiple repetitions for something to be procedurally encoded as an automatic sequence. This mode of pleasure seems closely affiliated with and even characteristic of music, but less so for speech, where emotions are more typically elicited by the listener’s relationship to the semantic meaning conveyed by the utterance. This paper argues that the difference in the appetite for repetition between musical and speech-based modes of communication is fundamentally linked with differences in the means by which these modes of communication elicit emotion. Margulis (forthcoming) explores this hypothesis in detail.

## Conflict of Interest Statement

The authors declare that the research was conducted in the absence of any commercial or financial relationships that could be construed as a potential conflict of interest.
